# Scaling up from sentience: modularity, conscious broadcast, and a constitutive solution to the combination problem

**DOI:** 10.3389/fpsyg.2025.1648930

**Published:** 2025-09-19

**Authors:** Thurston Lacalli

**Affiliations:** Biology Department, University of Victoria, Victoria, BC, Canada

**Keywords:** the hard problem, the combination problem, micro-consciousnesses, phenomenal consciousness, awareness

## Abstract

Complexity in biology typically has less complex evolutionary antecedents which, for consciousness, begs the question of how a fully elaborated and unified consciousness, as we experience it, would have been scaled up from what we can assume to have been simpler, or at least different, beginnings. This poses difficulties for some theories, but is much simplified if the contents of consciousness combine in a constitutive way, so the balance between contents can be adjusted by natural selection incrementally as required, across generations, in evolutionary time. This contrasts with theories postulating an integrative solution to the combination problem, and is easiest to conceptualize by supposing that conscious sensations arise from the action of modular entities, each of which, regardless of spatial location, contributes separately to the total experience. There are, in consequence, two very different models for consciousness: that it is (1) non-modular, non-local and fully integrated at a conscious level, the more conventional view, or (2) modular, local, and constitutive, so that integrative processes operating at scale are carried out largely if not exclusively in a non-conscious mode. For a modular/constitutive model that depends on a broadcast mechanism employing a signal, what may be most important is the amplitude of the signal at its source rather than how far it is propagated, in which case each module must be structured so its output has precisely controlled characteristics and adequate amplitude. A model based on signal amplitude rather than propagation over distance would still require that conscious sensations adapted to serve memory accompany cognitive functions over which they exert only indirect control, including language and thought, but fails to explain how a localized signal comes to be perceived as pervasive and global in character. In contrast, the problem with integrative models is the assumption that consciousness acts globally and only globally, which risks misdirecting attention, both in theory and experiment, to anatomical structures and neurophysiological processes that may have little to do with the processes by which conscious sensations are produced or how brains come to be aware of them.

## 1 Introduction

This is one a series of papers designed to address the problem of how consciousness would have evolved, with a focus less on specific scenarios than on evolution as a process and how that process constrains what we can and cannot suppose to have occurred. For the vertebrate lineage, there is increasing acceptance of the idea that consciousness originated among basal amniotes, reptiles perhaps, or early birds and mammals (Cabanac et al., 2009; Allen and Trestman, 2020), but no consensus regarding the nature of the first conscious experience, in other words, what it was “like” to have been the first vertebrate to be conscious. In accord with Feinberg’s usage (Feinberg, 2024), I will refer to this first occurrence of a conscious mental state as sentience, which must then explicitly incorporate an experiential component. Explaining how sentience is possible equates to Levine’s explanatory gap (Levine, 1983, 2009) or similarly, Chalmers’ hard problem (Chalmers, 1995, 2003) regardless of the qualitative characteristics of the experience itself. An emergent consciousness might, for example, have been no more than a faint glimmer of sensation affecting behavior in some very minimal way. The evolutionary trajectory would then have been from simple to complex, as contents of qualitatively different kinds were added to that first experience. Instead, sentience might from the start have included multiple forms of experience, which does not preclude them being combined together in quite different ways than they are today, in our consciousness. An ancestral consciousness might, for example, have comprised a set of ur-experiences, each incorporating some combination of sensations that to us would be distinguishable, but where the evolutionary process responsible for making them distinguishable (dimensional sorting, see Lacalli, 2022) had not yet occurred. Regardless of details, the real problem from the perspective of evolutionary process is to explain how it is possible for conscious experience to evolve incrementally so as to be adaptive on a continuing basis, scaling it up if need be, or changing the character of particular sensations, without disturbing the balance between contents, their contribution to the total experience, and the adaptive benefits of that total experience.

Which brings me to the combination problem, of understanding how separate kinds of experience are combined and unified in real time. This has philosophical aspects, mainly relating to panpsychism (Goff, 2006, 2009; see Montero, 2016 for the counterargument), but for neuroscience is primarily a mechanistic issue, as the analysis of resonance by Hunt and Schooler (2019) illustrates. Their formulation of the problem shows how complex a mechanistic explanation could potentially be, but it is nevertheless considered an “easy” problem in the sense that Chalmers uses the term because it can in principle be investigated using conventional scientific methods. This distinction between hard and easy problems is, however, too simple in my view, masking complications that depend on theoretical stance and mechanistic assumptions. There is, in particular, the question of whether the majority view of the combination problem, that it depends exclusively on integrative mechanisms operating in real time, is misguided in overlooking processes lodged in evolutionary time. By way of analogy, consider the role of the skeleton in controlling the motion of the body, which in real time depends on which muscles are active, but also on skeletal structure, which constitutively constrains what can and cannot occur. Comparable constraints for consciousness reside in the structure of the neural substrate, meaning structure in total, not just structure as it relates to neural connectivity, and it is a mistake to ignore structural features that may be crucial to the way the contents of consciousness are combined and unified when there is no valid justification for doing so. Of course we also want to understand the particulars of how and why consciousness evolved, but my intent in this account is a lesser one, of developing a framework for thinking about those particulars, while deferring a discussion of the results of that exercise to a future paper.

The account that follows explores the consequences of making two assumptions: (1) that consciousness in some way depends on a broadcast mechanism, and hence the production of a signal that is received and responded to, and (2) that it may be modular, in which case scaling up from one or a few modules would have been achieved simply by adding more modules. My use of the term module here is neuroanatomical and concerned with the neurons or neuronal assemblages that act as sources of conscious signals, which is different from modularity as it relates to cognitive function, either in general terms (Cosmides and Tooby, 1994) or to language processing more specifically (Robbins, 2017). There are both advantages and disadvantages to thinking about modules as sources of conscious signals, but one benefit is to highlight the importance of addressing the problem of spatial scale explicitly, regarding, for example, module size, the spatial range of the signal each might be supposed to produce, and where in the brain the various and sundry modules might be located. The cerebral cortex is where many suppose the neurocircuitry responsible for producing and responding to conscious sensations is to be found, but this is far from being a settled matter (Merker, 2007; Morsella et al., 2016, footnote 2; Earl, 2019), and the literature largely fails, in my view, to adequately reflect current uncertainty on this point. Careful attention is required also to explanatory structure and the role evolutionary arguments play in resolving the problems we face in explaining consciousness, whether classed as easy or hard. Hence I begin in section 2 with a survey of relevant theoretical issues, recasting them to a degree because that is what an evolutionary analysis requires, but in particular to show how the term “awareness” as I use it here should be understood in this context. Section 3 summarizes the conceptual problems that arise when thinking about how a modular consciousness might operate in practice, including the possible importance of amplitude at source to the causal efficacy of a broadcast signal if there are limits on how far it can propagate over distance. Section 4 then extends the analysis to specific contents, with a focus on vision. Vision is of particular interest in this regard because of what the experience of a conscious visual display says about the limitation imposed by the hard problem, that physical properties cannot be assigned to awareness, and whether that limitation is as absolute as generally supposed. Theater and concert hall analogies are employed at several points in the narrative, but this is not meant to imply that consciousness can be modeled as a stage performance for which the self is the audience, a stance to which Dennett (1991) has taken particular exception. The intent instead is simply to illustrate specific points that are important from an analytical perspective, and no more than that is implied.

## 2 The hard problem from an evolutionary perspective: existence and realization

**Figure 1:**
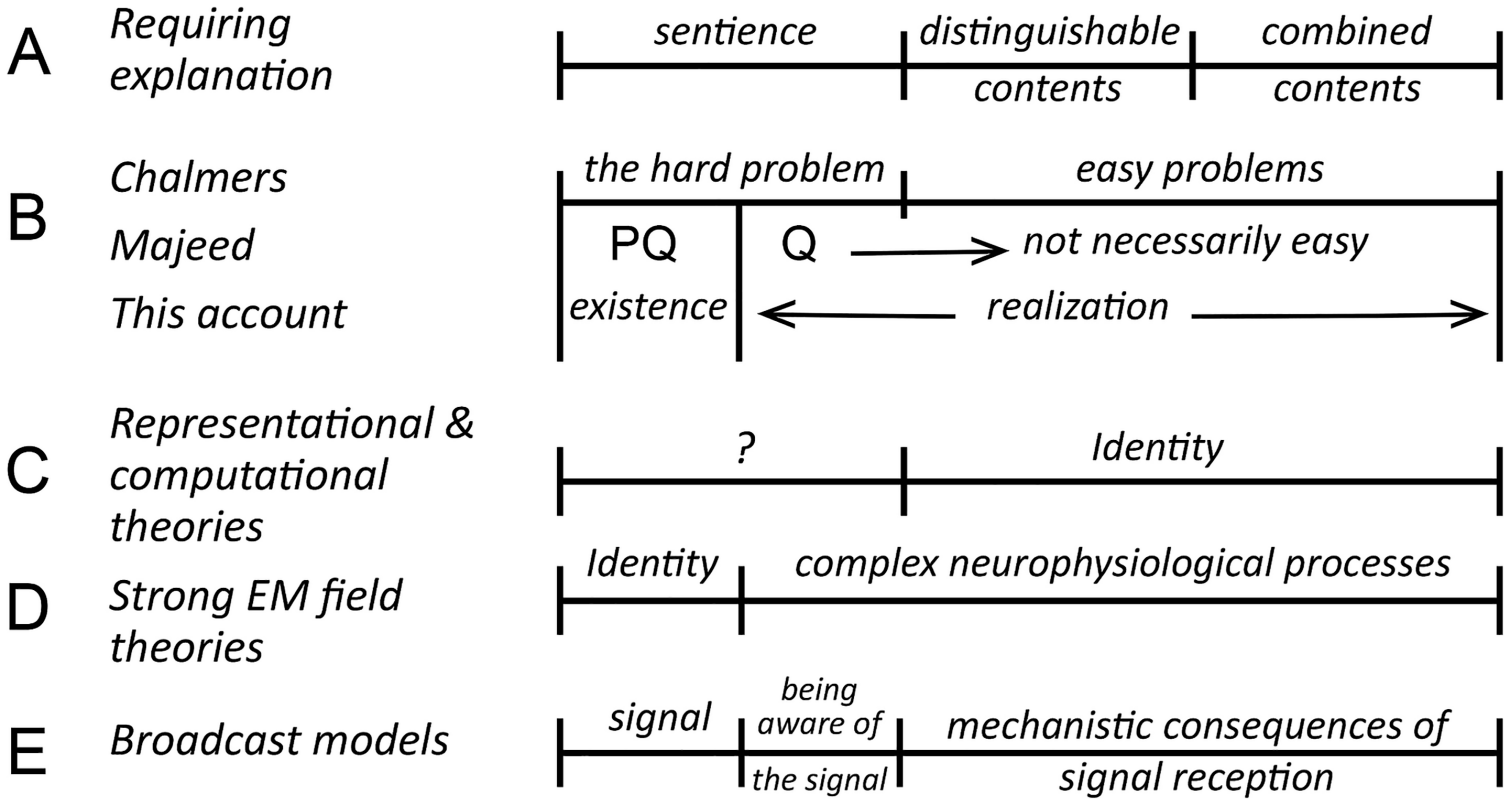
The hard problem in relation to neurophysiological and evolutionary questions concerning consciousness, a summary diagram showing how the elements of an explanation will align (along the horizontal axis) depending on theoretical stance. **(A)** The explanatory targets that any useful theory of consciousness needs to address, where the evolutionary point is that each depends on evolutionary innovations that require explanation: of how any conscious experience (= sentience) is to be explained, how distinguishable contents originate, and how they combine to produce the kind of consciousness we experience. **(B)** The hard problem from three different points of view. For Chalmers (1995) it equates to explaining how sentience can exist. Everything else about consciousness is then assumed to be explainable in conventional scientific terms, and hence easy in principle. The refinement suggested by Majeed (2016) is to explicitly include unspecified aspects of the way consciousness is realized by biological brains in the hard problem (his “thus and so” of conscious experience) where, depending on theoretical stance, the transition from what is hard to explain to what is easy may differ. For my purposes in this account, which is primarily evolutionary in focus, a more useful distinction is between the problem of existence, which is ontological, and realization, which encompasses all that evolution has achieved in order for biological brains to manifest what ontology makes possible. The dividing line between the question of existence and realization is then quite specific, being the point at which evolution enters as a causal influence. **(C,D)** Two examples of how different theories deal with these explanatory targets. First, that representational and some computational theories, with their focus on sensory processing, suppose that the content of the process, or what it represents, equates to the resulting conscious experience. The combination problem is then solved by an identity, an assertion that must be judged on its own merits, and which may, or may not, depending on interpretation (hence the question mark) be supposed to solve all or part of the hard problem. Strong electromagnetic (EM) field-based theories adopt an opposing strategy in asserting that the problem of existence is solved by an identity, so that consciousness would be embodied in some way in an EM field structure generated by neurons. This leaves the problem of accounting for everything else about consciousness, though easy in principle, potentially quite difficult in practice (Hunt and Schooler, 2019). **(E)** My interpretation of how broadcast models of consciousness would fit into this framework, that explaining sentience and the hard problem has both an ontological component, relating to the signal and its source, and a biological/neurophysiological one of how first-person awareness of the signal is achieved. Everything else about the response to the signal is easy by comparison, and in principle soluble by a suitably detailed understanding of the connectomal and extra-connectomal mechanisms involved.

Theories of consciousness are diverse in mechanistic terms, but also in how each deals, or fails to deal with the hard problem (Chalmers, 1995; Atkinson et al., 2000; Seth and Bayne, 2022). For Chalmers what is hard in a foundational sense is the question of how conscious experiences of any kind are possible, but from an evolutionary perspective this is bound up with the problem of understanding the evolutionary innovations required to turn the potential for a conscious experience into reality. It is useful then to separate the problem of existence, the ontological question of how any kind of conscious experience can exist, from the evolutionary one, of how the emergence of consciousness in biological brains is to be explained, which I refer to as the problem of realization (Figure 1). Both are required to explain vertebrate consciousness fully, meaning that we must account for sentience as it first evolved, but also for a consciousness consisting of distinguishable contents combined in particular ways (Figure 1A). How those elements of the explanation align with Chalmers’ distinction between hard versus easy problems, and Majeed’s recasting of the problem, is shown in Figure 1B. PQ in Majeed’s formulation (Majeed, 2016) is the question of how consciousness of any kind can exist, while Q is the problem of explaining the “thus and so” of consciousness, defined by the author as “the precise nature of the relationship between such [generative] processes and our phenomenally conscious experiences.” Q for a biologically-realized consciousness is then implicitly evolutionary in its focus on those features of consciousness that have been realized by our brains, which are conscious because of evolution, where what is hard versus easy to explain may depend on theoretical stance.

So, for example, consider representational theories (Gennaro, 2018) or computational ones (Sun and Franklin, 2007; Stinson, 2018), for which consciousness is supposed to arise from a processes, basically computational or algorithmic in character, set in motion by the connectome (the spike code of Hunt and Jones, 2023, or dependence on computational functionalism in the terminology of Gomez-Marin and Seth, 2025), irrespective of other physical effects resulting from neural activity. There are objections one can make in principle to theories of this kind, especially where a causal role is ascribed to information processing (Manzotti, 2012; Wood, 2019), but what unites them is equating a large-scale entity, a computation or a representation, to the conscious experience that results. This solves the combination problem through an identity, as indicated in Figure 1C, but without explaining how this is achieved at a mechanistic level. Chalmers’ argument is that the hard problem cannot be solved by assertions of this kind, even in principle, hence the question mark in Figure 1C. But proponents of such theories may disagree, claiming that they do, and this is accommodated in Majeed’s formulation because the reach of the hard problem is extended to include such claims. In contrast, consider the problem faced by electromagnetic (EM) field-based theories in their strong form, meaning reductionist variants (Hunt and Schooler, 2019), where the source of consciousness is attributed to EM field structure. This again postulates an identity (Figure 1D), but on the existence side. Solving the hard problem is then a matter of accounting for this identity, both why it is possible and how it is implemented, where the latter equates to the core issue for realization, of how neurons convert EM field effects into first-person awareness of those effects. If this depends on integrative processes operating on a large scale, it cannot in my view be an easy problem even in principle.

Grounding such theoretical concerns in established physical and biological principles requires in a narrower focus and a better defined set of assumptions. To this end I will restrict the analysis that follows to broadcast models of consciousness (Figure 1E), where the assumption is that we are dealing with a mechanistic signal of some kind that has properties similar to more familiar kinds of broadcast in originating from a source, having a means and medium of propagation, and a defined spatial range. This approach has its own limitations, in depending on conventional physics to explain a situation where conventional physics may not apply, but it is the only way I see at present to explore the problem of how a complex consciousness evolved in a biologically realistic setting. A note is required here on terminology, that following Chalmers, I will use “experience” or “experiential” to refer to anything experienced consciously, whereas “consciousness” is used in a general way to for the sum total of all that the conscious pathways in the brain do and make possible. So, for example, in a signal/response model (Figure 1E) the hard problem of explaining consciousness would have two parts, of explaining in ontological terms how a signal capable of being consciously perceived can exist, but also, and separately, how biological brains make it possible for an animal like us to have a first-person awareness of that signal. Because the mechanism by which awareness is achieved by brains is unspecified, and indeed unknown, it then becomes the part of realization that resides with the hard problem. In consequence, mechanistic assumptions concerning the nature of the signal do not apply to awareness, which is a separate phenomenon whose physical properties, if it has any, lie outside those assumptions. It should be evident also that the conceptual framework developed here could be generalized to deal with any entity one might choose to suppose is responsible for consciousness, whether this involves a broadcast mechanism or something else. An example is the self as defined by Merker (2013), which in his formulation is required for sensory representations to be transformed into conscious contents. The same questions I pose in the next section regarding conscious broadcast would then apply equally to the nature and properties of such a self, as to whether, for example, it is to be conceived of as a modular entity or a component of a larger-scale integrative process.

## 3 Modularity, and a constitutive solution to the combination problem

**Figure 2:**
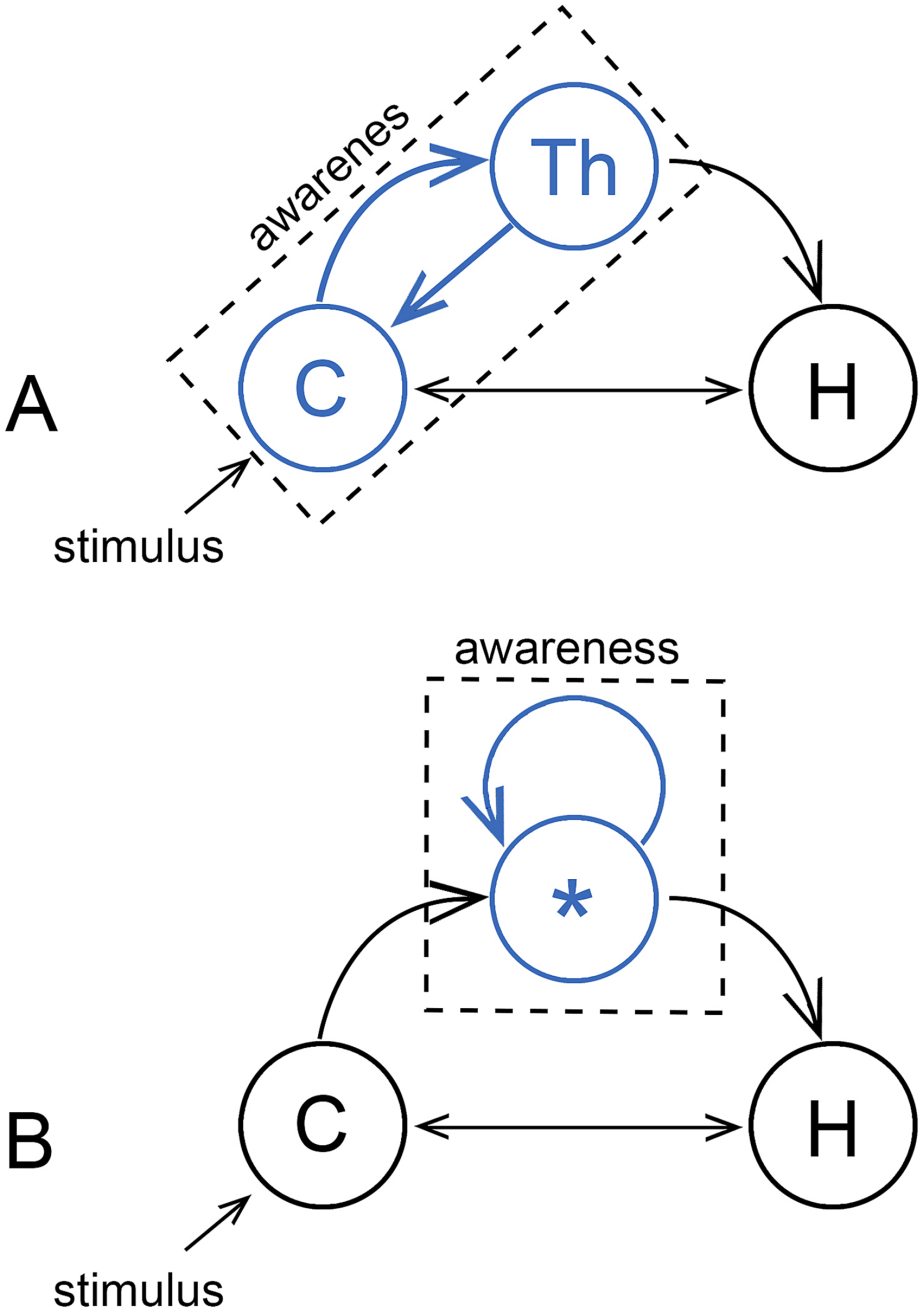
Two models for consciousness, with the hippocampus (H) shown as a target on the assumption that consciousness acts primarily to serve memory. **(A)** The majority view, that consciousness is realized on a large scale through a cooperative interaction between the cortex (C) and thalamus (Th), where the source of conscious sensations (indicated in blue) is either the cortex acting alone or in combination with the thalamus. Awareness then arises from large-scale integrative processes. The alternative **(B)** is a modular consciousness where each of the individual modules, only one of which (*) is shown, produce conscious sensations (again, in blue) via localized neural activity unspecified as to location. The individual modules could then be physically small, so if we chose to represent each module as a blue dot, there could be many such dots within the confines of the circles representing the cortex in **(A)**, or the thalamus, or both. Whether consciousness can then be integrative or not depends on the spatial range of whatever causal effects each module is able to produce, whether large or small, the consequences of which are outlined in the next figure.

Consciousness can be supposed to arise from neural processes in two ways: (1) from large-scale patterns of neural activity involving neurons dispersed widely across the neural substrate, generating in some models a global brain state that maps to the resultant experience, or (2) as the summation of the individual contributions of subsidiary modules, each capable on its own of generating a conscious experience. There are then two corresponding ways of thinking about how an emerging consciousness, operating first on a small scale, would have been scaled up by evolution: (1) from a single integrated construct that began small and subsequently became larger, or (2) by adding modules as required. The first option corresponds with the idea that “consciousness is ‘big’” in the words of Blumenfeld (2023), meaning indivisible and dependent on cortical and cortico-thalamic processing on a large scale (Figure 2A). This accords with models of cognitive function that feature large-scale networks (Bressler, 2008; Petersen and Sporns, 2015; Ito et al., 2022), linked by coordinated patterns of activity, waves and resonance effects of various frequencies and strengths (Varela et al., 2001; Sauseng and Klimesch, 2008). From an evolutionary perspective, this would imply that consciousness has in some sense always big even if, as it first evolved, the part of the brain hosting it was physically quite small. The second option, the modular one, implies a consciousness assembled from subunits that are and have always been small in relative terms (Figure 2B). This is akin to the micro-consciousness proposal by Zeki (2003), developed to explain the contribution of separable components of visual processing to visual experience. There is the related question of how large a fragment of functional cortex is required to sustain consciousness, whether in a damaged brain or in neural organoids (e.g., see Bayne et al., 2020 on islands of consciousness), but more relevant to normal brain function in my view are observations by Berridge and Kringelbach (2015), who attribute sensations of pleasure (of liking rather than wanting) to subcortical centers of neural activity those authors refer to as hotspots. Whether the hotspot concept is valid for a wider range of conscious sensations remains to be determined, but if it were, only half the problem is solved, of where sensations are produced, not where they are responded to, which in the hotspot model is unspecified.

**Figure 3:**
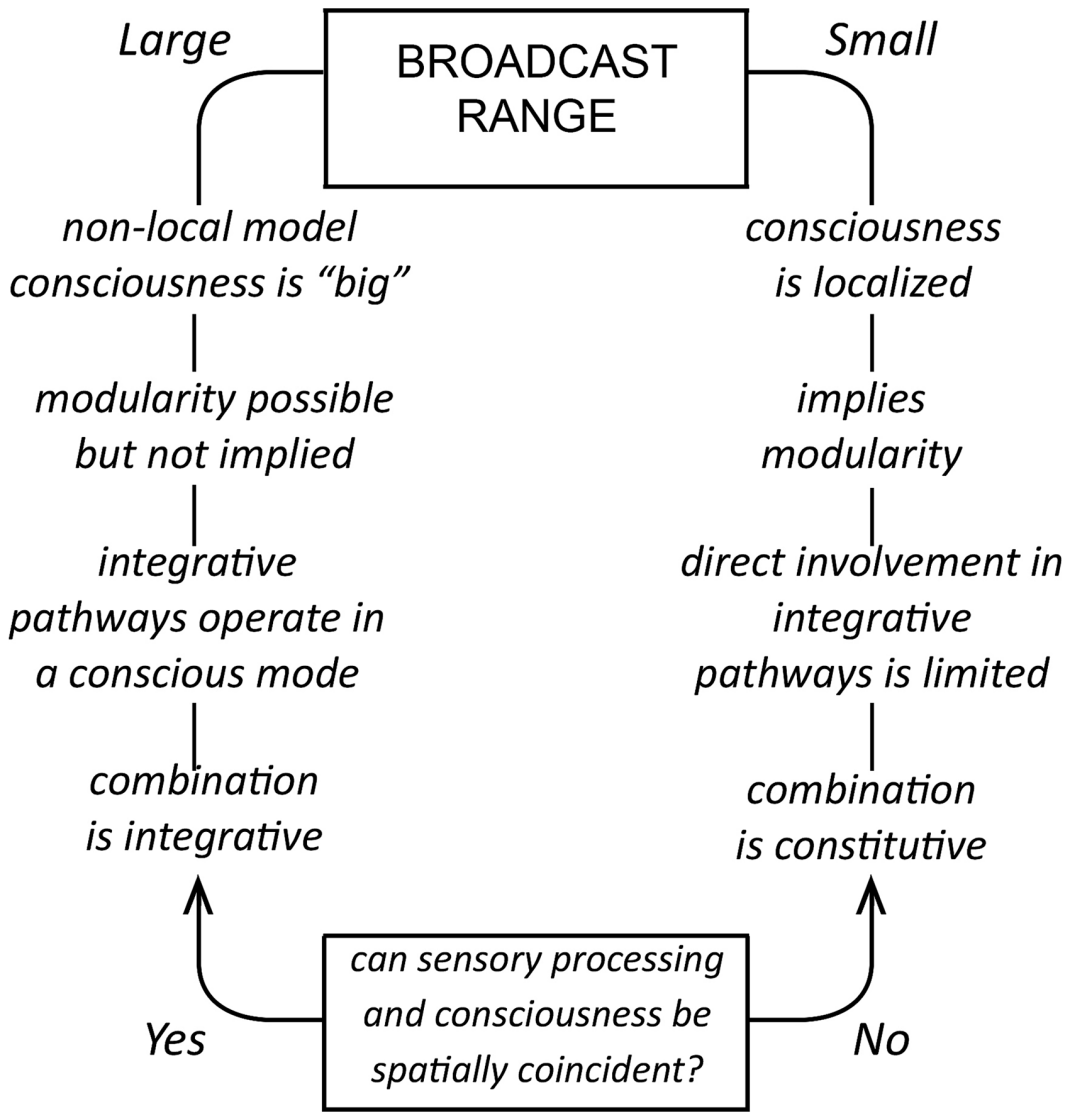
For a broadcast model, how the supposed range of a mechanistic signal impacts the way consciousness is structured and operates; a summary of the main line of argument in the text. If the spatial range is large (on the left), the signal can exert its influence across a large fraction of the neural substrate and participate directly in integrative activities operating at scale. Consciousness can then be “large” in being unified at that scale. For a signal of limited spatial range (on the right), as might be generated by a modular consciousness, integrative activities beyond the range of the signal would necessarily carry out their designated functions in a non-conscious mode, receiving conscious inputs at one remove via non-conscious pathways originating from centers the signal is able to affect directly. The combination problem for this case cannot then be solved in an integrative fashion in real time, but relies on evolution to devise a constitutive solution of proven adaptive utility, tested across generations. The issue the figure does not address is the spatial range of awareness. That is, if neurons respond to a localized modular signal, we may assume that they are located in or close to the module. It is then their response that makes us consciously aware of the signal, but whether awareness itself is likewise limited in its spatial range is not specified. It is at this point that we confront the hard problem, that mechanistic assumptions do not tell us about the properties of awareness, for which limitations conceived of in mechanistic terms may or may not be meaningful.

The idea that consciousness depends on a broadcast of some kind is widely used as a conceptual device for both top-down theories like global workspace theory, and bottom-up ones based on EM field-based effects (Kitchener and Hales, 2022; Hales and Ericson, 2022). It is in some cases no more than a metaphor, but if taken literally, would require a signal and a target for that signal, neurons in this case, that monitor (see Irwin, 2023, on the monitor function) and respond to the signal in a non-epiphenomenal way. To account mechanistically for conscious integrative processes acting at scale, we would require a signal with a sufficiently large spatial range to affect non-conscious neural processes operating some distance from the source of the signal. Otherwise, for signals of limited range, as outlined in Figure 3, a direct role for the signal in such processes is precluded. Conscious inputs would then be acting one remove, via synaptic pathways operating in a non-conscious mode, which accords with the argument that sensory processing in the cortex is likely in any case to be carried out in subjective silence (Merker, 2007; Earl, 2014, 2019). There is a complication, however, because for any generative signal we choose to postulate, we cannot assume an identity between the effects of the mechanistic component, meaning the signal and the proximate response to that signal, and effects arising from being consciously aware of those events having occurred. Awareness could be different in having a different spatial range, or requiring other neurons, or other mechanisms, to be fully realized. Hence the caveats in the legend to Figure 3 and elsewhere in this account, and the difficulty of making a convincing argument on mechanistic grounds alone when the specter of the hard problem is lurking in the background. But I will make the attempt nevertheless, the point being to show that consciousness can be explained without requiring action over distance on the part of either the signal, or awareness of the signal, if the combination problem is solved constitutively.

Consider first the case of a conscious broadcast mechanism dependent the neural connectome. Given that axons can be arbitrarily long, there should be no limit to the distance over which the broadcast could exert its effects. If this depended waves of neural activity propagated over distance, for example by having the neurons act as coupled oscillators, the propagated signal could also be both enhanced and materially changed in character as it propagates. The difficulty comes with a signal transmitted by other mechanisms, meaning extra-connectomal ones, which could include paracrine transmitter release or EM field effects. The latter are generally supposed to be propagated by ephaptic coupling (Hales and Ericson, 2022; Hunt and Jones, 2023), which occurs *in vivo* (Han et al., 2018; Ruffini et al., 2020), but is considered too weak or to propagate too slowly to be of use for any but a few specialized purposes (Weiss and Faber, 2010; Anastassiou and Koch, 2015). Part of the problem is explaining how an ephaptic signal would be propagated across a heterogeneous and highly structured neural substrate simultaneously engaged in other activities, all of which generate EM fields that are potential sources of interference. This problem is avoided by assuming propagation is largely irrelevant, which it would be for a signal of limited range. The key point here is that there are two ways a signal can be “big” for any wave-based model: by being propagated over large distances, which assumes a medium and a mechanism of propagation, or by its amplitude at source being large irrespective of whether it is propagated or not. And if not, as may be the case for an EM field-based mechanism, it would be the magnitude of the effect generated within the confines of the module that matters. I develop this example more fully in the next paragraph, but the argument can be extended to a wider range of theories, including those not dependent on EM fields, so long as there is a mechanistic component that is suitably signal-like to which something corresponding to amplitude can be assigned.

To clarify the issues involved in thinking about the constitutive features of a broadcast mechanism dependent on amplitude, an analogy is useful: of the sound produced by a symphony orchestra. An orchestra consists of diverse instruments producing sounds that depend in each case on the physical shape and resonant properties of the instrument in response to being struck, plucked, or blown into. Physical shape and resonant properties then operate as boundary conditions for a set of acoustic waves that depends on the choice of instruments, whether there are say, 10 flutes for each cello or 10 cellos for each flute, and how loudly the score specifies each is to be played. These are constitutive features with respect to an individual performance because they are in place before the concert starts and are unchanged as it progresses. How audible a particular instrument is to the audience will depend on how loudly it is played and the distance involved, which means that both amplitude at source and spatial range are important where, for acoustic waves, these are codependent. In contrast, for a modular consciousness dependent on a signal of limited range, everything relevant to the magnitude of the overall effect would occur locally and depend on amplitude at source.

This could have important consequences if the signal is EM field-based. Amplitude in that case would depend on current density, a measure of ion movement across membranes for all such events within the confines of the volume in question, the module in this instance, and the spatiotemporal coherence of the fields those events produce (Pockett, 2012; Hales and Ericson, 2022). If the output of the module depends on synaptic input to it, it may well have particular frequency characteristics dependent on that input, but at the same time we require a 3-dimensional configuration of the various cellular structures involved that allows the fields due to ion flow to sum rather than canceling out. The task for evolution in this case, as succinctly put by Colin Hales, who concurs on this point (pers. communication), would be to “contrive cell densities and connective architectures to the extent needed” to produce a field with the required characteristics. Which would also require that there were developmental mechanisms in place to ensure that each module is precisely structured so that current flows are optimally aligned. A prediction of the constitutive model therefore, at least in its EM field-dependent version, is that there should be morphological correlates of consciousness in addition to activity-dependent ones, in that assemblages of neurons involved in producing conscious sensations would be configured differently at scales below the connectomal compared with those that are not, which could involve, for example, the spatial ordering of dendrites and synapses along those dendrites (Lacalli, 2020).

To return to the orchestral analogy, a problem for a modular/constitutive model of consciousness is to identify the neurobiological counterpart of the audience. If we are dealing with a broadcast signal of limited range, the audience for that signal would necessarily also be localized, to each module and its immediate vicinity. This presents a conceptual challenge, of explaining the global character of our awareness of conscious experience if the signals responsible have no direct physical effects much beyond the bounds of the modules that produce them. But now the hard problem makes an entry because, without a physical model for awareness, we have no understanding of how the brain becomes aware of anything, globally or otherwise. Hence, for a modular/constitutive model of consciousness it is within the bounds of existing theory to suppose that awareness of a signal-generating event does not depend on its being broadcast over distance, but is possible simply by virtue of the event having occurred. It is as if, for the orchestral analogy, the instruments collectively constitute an audience where it is sufficient that each hears only the sounds that it has itself produced. While this is hardly credible for instruments made of wood and metal, the “instruments” in the case of consciousness are modules assembled from neurons, so it is neurons and only neurons^1^ that are ultimately responsible for awareness, regardless whether this occurs at a large or small scale. And so, whether the argument that awareness acts in a causal way only locally appears, on the face of it, to be credible or not, there are no scientific grounds for rejecting it when we have no clear understanding of what awareness is, its properties, or the physical laws it obeys. This illustrates what I will refer to as the no-free-lunch principle, a key part of the argument made by Chalmers (1995, 2003) to the effect that mechanistic (more generally, reductionist) assumptions deliver no more than mechanistic (reductionist) insights, and so tell us nothing about how the hard problem, to which awareness belongs, is to be resolved. I return to this point in the next section in relation to an example, involving vision, where the principle may be violated.

A second problem for a modular/constitutive model relates to epiphenomenalism, that for any function carried out beyond the range of a modular signal source, we need not require that awareness is causally efficacious with respect to that function if it can be accounted for through indirect effects that act at one remove. If we further suppose that consciousness evolved first and foremost as a servant of memory, its role in other cognitive processes would be due to the dependence of those other processes on consciously-encoded memories. The model for this derives from Libet’s experiments (Banks and Pockett, 2007; Budson et al., 2022) where, despite disagreements on interpretation (cf. Neafsey, 2021; Triggiani et al., 2023), the time delay between the initiation of an action and conscious awareness of the intent to do so can be explained by having conscious inputs act through memory to adjust predetermined sequences of non-conscious actions already in progress. Superficially this makes it appear that consciousness is epiphenomenal with respect to those actions, but a more accurate characterization is that it is only pseudo-epiphenomenal due to the time delay required for memory access. This brings up a second point, that tracking non-conscious processes with a pseudo-epiphenomenal overlay of conscious sensations, or a series of conscious states, provides a mechanism for reinforcing conscious links to memory on an ongoing basis. This would imply that, for the conscious component of memory, as with any brain function dependent on synaptic plasticity, there is a risk of degradation over time. Hence the utility of having a mechanism in place whereby, through repetition, links to specific memories are reinforced to embed them more fully in the cognitive processes that depend on them. This is consistent with the supposition that, while the process of learning a particular task through a conscious mechanism may have an endpoint, keeping the learned state fully operational on an ongoing basis does not, a point relevant to the discussion of language in the next section.

A final problem for the modular/constitutive model is that, absent an arena in which the various broadcast signals interact directly, we would require some other explanation for why different sensory modalities should be experienced in particular and qualitatively different ways. This is because, if the broadcast signal acts only at the initial step in a causal sequence, and so long as the integrative process occurs later in that sequence, information about the experiential character of the initiation event is irrelevant to those later steps. There are several points to consider here, first that it could be that frequency information specific to each modality is part of the mechanism used for encoding the source of a conscious input at the level of the hippocampus (see Tacikowski et al., 2024 for its frequency dependence). Conscious signals should then, at the very least, differ in their frequency-dependence, so they could not be identical. But this fails to explain, in a positive way, why they should differ in particular ways that are adaptive. Why, for example, would light as we perceive be selected as the optimal way of constructing a conscious visual display rather than some other quale? Selection implies competition, which could be a matter of controlling attention. If this depended on a cognitive workspace of some kind, the selection process could be limited to mediating between conflicting inputs through selective attention rather than being integrative in a broader sense. But there would still the question of how such a workspace could incorporate a conscious component for the case of conscious signals that cannot exert their effects much beyond the boundaries of the modules that produce them. There would appear to be two options here: that (1) local interactions between modules or groups of modules and their nearest neighbors are sufficient, when summed, to provide the required degree of integration, or that (2) we are again dealing with a consequence of our inability to make informed judgments about how the properties of awareness relate to those of the signal that is its proximate cause. Having greater spatial range might be one such property, but there could be others, and this, in my view, is the nub of the problem when it comes to understanding awareness, and the most promising route for investigation going forward. The issues are, however, sufficiently complex as to require a more extended treatment than is possible here.

As to what this all means in practical terms, a modular model highlights the potential importance of focal centers of activity in the brain as sources of conscious signals, where the task for the experimentalist is to discover where these are and how they function. The work by Berridge and Kringelbach (2015) provides in my view a useful reminder of how much we have yet to learn on this topic, and their conclusions, should they apply more broadly, have interesting implications. One relates to energy cost, that whatever the broadcast mechanism, the level of synaptic activity required to produce a signal will impose costs of one kind or another. For conscious contents present in awareness on a continuous basis, vision being an example, the prediction is that selective pressures would act to minimize those costs. For an EM field-based model, energy cost scales with current density, meaning ion flows per unit volume, and so could be reduced by reducing the size of each module. Modules should then be physically as small as possible, which could make them difficult to identify and record from. A further complication for all of the above arguments is the point raised by Polák and Marvan (2019) that there may be phenomenal states able to affect behavior in the same way as conscious states, but without their conscious component. If mechanistically similar, then in practice, distinguishing experimentally between conscious and non-conscious broadcast centers could be a non-trivial problem, and it is premature to speculate on how it might be resolved.

## 4 Complex contents and the “structure” of consciousness

The analysis so far as been cast in a way that makes it relevant to the simplest forms of experience, which one would normally construe as meaning phenomenal contents on the assumption that these evolved first. Whether true or not, it is a separate set of questions as to how qualia, once they evolved, or perhaps as they evolved, were co-opted to perform more complex cognitive functions. Rather than the usual distinction made here, between phenomenal and access consciousness, which can be problematic (Overgaard, 2018), I prefer to distinguish between contents that cannot be deconstructed or analyzed and those that can. The former are then by definition fundamental, structureless, and ineffable units of subjective experience, i.e., qualia, whereas contents more complex than a quale, formats in my terminology (Lacalli, 2021), have additional features that must be accounted for by other means. Our species has two contents that clearly conform to my definition of a format: vision, which is structured to display the external world in relation to a fixed viewpoint (Merker, 2007, 2013), and language (Jackendoff, 2002; Pinker and Jackendoff, 2005) including everything that flows from the use of language, thought, and the inner voice that accompanies thought. For animals that are conscious but do not use language, vision and visual memory presumably take its place as a cognitive resource and for constructing narrative which, based on the reported experiences of members of our own species who think visually (e.g., Grandin, 1996), are tasks conscious vision is entirely capable of performing. There may be other formats, the mapping of mechanosensory experience through referral being an obvious example, but restricting the argument to vision and language avoids complications and highlights my main point, of the differences between percepts that rely on sequential processing and those realized as a simultaneous display.

The first point relates to structure, not the internal structure of vision and language referred in the previous paragraph, but of consciousness itself. Assume provisionally that it is correct to characterize our consciousness as combining phenomenal contents and two formats. This is then its structure, which defines the way we experience external reality when awake, as a continuous visual experience combined with an overlay of language and thought into which phenomenal experiences intrude in response to specific stimuli. But why this structure rather than some other? By analogy to the vertebrate skeleton, whose flexible core reflects our aquatic origins as undulatory swimmers, formats could be thought of as equating to appendages that evolved secondarily as additions to an existing core of phenomenal contents. But is this an accurate characterization, and even if it is, could there have been other outcomes, and different ways dealing with the various sensory modalities? Rather than a conscious 2-dimensional visual display, for example, suppose olfactory or somatosensory information were displayed in that fashion, leaving visual stimuli to be experienced episodically, like touch, only when a response mediated by consciousness was required. And why have multiple formats in the first place? One might suppose that presenting sensory information to a conscious self in more than one format would be a way of minimizing interference compared with requiring all sensory inputs to compete on an equal basis, on the same stage so to speak, as phenomenal contents do. For the latter, relative amplitude governs attention, meaning who is shouting the loudest, and there will be limits as to how may competing voices can be attended to at any one moment. There is also the question of why there are two formats rather than some other number, where it may not be a coincidence that visual information, displayed in a spatial mode, and language, experienced as a temporal sequence, exhaust between them the options available for using space and time as format-specific ways of mapping sensory information. The conjecture would be that the structure of consciousness at this level is less a reflection of evolutionary contingency, that there happen to be two formats, than of an ontological limit, that there can only be two,^2^ and the task for evolution is to discover how to use them to best advantage.

The question of interest is then whether scaling up consciousness from simpler beginnings builds on mechanisms already in place or requires additional evolutionary innovations and novel mechanisms. For language, because it operates as a time sequence, there is the option of coopting and reconfiguring pathways already involved in the control of action sequences in response to auditory inputs, which accords with the supposition that speech and language have their origins in gestural control (Arbib, 2005; Gentilucci and Corballis, 2006; De Stefani and De Marco, 2019). The interventionist role conscious inputs play in the control of action sequences could then provide a model for the role conscious inputs play in directing progress through a sequence of linguistic states. Extended to encompass abstract thought, for most of us a verbal construct, the inescapable conclusion is, in the words of G. A. Miller (cited by Morsella et al., 2016, p. 14): “that it is the result of thinking, not the process of thinking, that appears spontaneously in consciousness.” Or further, taking habitat navigation as a model for the intervention sequence (Lacalli, 2024), that our proportionately much larger brains compared with other terrestrial vertebrates reflects the fact that we routinely navigate, through language and thought, a much larger “landscape” than those other species.

Dealing with conscious vision is more problematic because the way it is displayed, as a 2-dimensional perceptual map, is unique to that modality. This form of display could have been constructed piecemeal over an extended period of evolutionary time, perhaps from simpler beginnings as Merker (2007, p. 72) has suggested, where “targets might appear as mere loci of motion in an otherwise featureless noise field.” Vision might well in fact have functioned for quite a long period of vertebrate history in an unconscious mode, with a conscious component being added only secondarily as forebrain structures became increasingly involved. But at some point, it would appear, an event occurred that allowed conscious mechanisms operating in a temporal sequence to produce a visual display perceived as a single entity on a moment-to-moment basis. Explaining how this was done poses, in my view, a uniquely difficult challenge for any integrative theory of consciousness. In contrast, for a modular/constitutive model we need only to extend the idea of sensation-generating hotspots, replicating them in sufficient numbers to account for the individual point-by-point elements of the display. Mapping the output of each module to a corresponding point on a perceptual map is a simple idea that, if experimentally validated, could have deep implications not only for understanding signal production, but awareness as well. It would mean that changing the position of a module in real space (its absolute position) or relative to other modules (its relative position) has consequences in perceptual space, demonstrating that awareness either is or can be made to be position-dependent. This represents specific information about the properties of awareness that philosophical arguments would suggest are in principle beyond our reach. In answer to this apparent conundrum, I would observe that by adopting a modular model we make an implicit assumption that modules are distinct physical entities, and as such no two can occupy the same physical space. Perhaps it is this requirement that imposes a corresponding degree of order at the perceptual level, but if so, it is not clear to me precisely why that should be. There nevertheless does appear to be a potential reward here, of the proverbial free lunch should a modular/constitutive model be proven correct, paid for by the hard work evolution has done in contriving a mechanism whereby, for vision, the correspondence between physical and perceptual space is evident by inspection.

## 5 Conclusion

This account distinguishes between two very different ways of explaining how a unified consciousness combining diverse contents is generated. The more widely accepted view, the integration consensus (Morsella et al., 2016), supposes this to be a consequence of integrative processes operating in a conscious mode on a large scale. The alternative is to suppose that consciousness is a modular construct, where the total experience is simply the summed output of those modules active at any given moment in time. If this involves some kind of broadcast signal, then the spatial range of such signals becomes an issue if, because of limited range, they cannot play a direct role in integrative processes operating at scale. The cortex would then be unlikely or unable to operate in a conscious mode, but there are plausible arguments to be made that it does not do so in any case, notably by Merker (2007) and Earl (2014, 2019). Theirs are minority views, and also run counter to our intuition that a consciousness like ours is necessarily “big” and so should depend on mental states occupying a large fraction of the neural substrate. But it is precisely on this point that the plausibility of a modular/constitutive model of consciousness hinges: that thinking of consciousness as “small” may be counterintuitive, but cannot be ruled out without a scientific justification for doing so, which would require knowing more about the physical properties of conscious awareness than we do. This then leaves us largely in the dark on the question of how much of the brain and what parts of it are required for us to be aware of the sensations our brain generates, and how many neurons of what kinds this might require.

The idea of a modular, constitutive consciousness, generated by isolated neuronal assemblages, may seem a piecemeal and inelegant solution to the problem of constructing a unified consciousness, far from an optimal design from an engineering standpoint. Inelegant, *ad hoc* solutions are, however, entirely typical of evolved systems and evident at all levels, from the anatomical to the genomic (Lewin, 1984; Akam, 1989). Criticizing the modular/constitutive model for failing to conform to our preconceptions as to how the conscious control of behavior *should* operate lacks force. But a problem for the modular/constitutive model is to explain why consciousness should accompany functions such as thinking and decision-making that it does not directly control. This is not a question of epiphenomenalism in a philosophical sense, but of what I have referred to above as pseudo-epiphenomenalism, where conscious sensations act at one remove, through memory. Hence the conclusion of this account, that a modular/constitutive model is both mechanistically possible and theoretically justifiable, but leaves higher order cognitive processes that depend on memory to operate largely if not exclusively in a non-conscious mode.

This then provides rationale for seeking neural correlates of consciousness that are causally determinative in brain structures other than cortex, and for patterns of activity that may differ from the propagated waves of activity that tend to attract the attention of most laboratories currently investigating this issue. The modular/constitutive model instead directs attention to focal centers of activity and to neuronal cell types that may have distinctive morphological features that change in observable ways during the process by which conscious agency is learned in each generation. For visual, acoustic and mechanosensory pathways I would suggest, taking my cue from Merker (2013), that the thalamus and its various nuclei are structures deserving particular attention, along with the subcortical amygdala for affect and olfaction. Pleasure on the other hand is somewhat of an outlier in being associated with a combination of dopaminergic projections and sensations generated in limbic centers (Berridge and Kringelbach, 2015), implying a separate anatomical basis and perhaps a separate origin. I conclude from this that vertebrate consciousness may be divisible into parts, perhaps three, like Gaul, or perhaps more, each of which may need to be investigated and understood on its own terms.

As a final point, it should be mentioned that a modular model says nothing specific about how small a given module might be, and hence accords with the idea that consciousness could be realized by brains far smaller than those of vertebrates, and perhaps even by individual cells (e.g., see Baluška and Levin, 2016; Baluška and Reber, 2019; Edwards and Somov, 2023) and Robinson et al. (2024) for the counterargument. This is an extension of the modular model that I choose specifically not to make here, as it goes well beyond the more limited goal I have set myself in this account: of examining modularity as it might apply to vertebrate brains specifically, for the insights that provides into the innovations required for vertebrates to evolve consciousness. There we are on much firmer ground, with a real prospect of devising convincing experimental tests to either confirm or reject the modular model.

## Data Availability

The original contributions presented in the study are included in the article/supplementary material, further inquiries can be directed to the corresponding author.

## Footnotes

^1^Which is not to rule out the possible involvement of glial cells.

^2^Again, to simplify the argument, I defer consideration of a third possibility represented by the topographical somatosensory map.

## References

[ref1] Akam M. (1989). Making stripes inelegantly. Nature 341, 282–283. doi: 10.1038/341282a0, PMID: 2797143

[ref2] Allen C. Trestman M. (2020). “Animal consciousness” in The Stanford encyclopedia of philosophy ed. Zalta E. N. (Stanford, CA: Metaphysics Research Lab, Stanford University). Available online at: https://plato.stanford.edu/archives/win2020/entries/consciousness-animal/

[ref3] Anastassiou C. A. Koch C. (2015). Ephaptic coupling to endogenous electric field activity: why bother? Curr. Opin. Neurobiol. 31, 95–103. doi: 10.1016/j.conb.2014.09.002, PMID: 25265066

[ref4] Arbib M. A. (2005). From monkey-like action recognition to human language: an evolutionary framework for neurolinguistics. Behav. Brain Sci. 28, 105–124. doi: 10.1017/S0140525X05000038, PMID: 16201457

[ref5] Atkinson A. P. Thomas M. S. C. Cleeremans A. (2000). Consciousness: mapping the theoretical landscape. Trends Cogn. Sci. 4, 372–382. doi: 10.1016/S1364-6613(00)01533-3, PMID: 11025280

[ref6] Baluška F. Levin M. (2016). On having no head: cognition throughout biological systems. Front. Psychol. 7:902. doi: 10.3389/fpgyg.2016.00902 27445884 PMC4914563

[ref7] Baluška F. Reber A. (2019). Sentence and consciousness in single cells: how the first minds emerged in unicellular species. BioEssays 41:e1800229. doi: 10.1002/bies.201800229 30714631

[ref8] Banks W. P. Pockett S. (2007). “Benjamin Libet’s work on the neuroscience of free will” in The Blackwell companion to consciousness. eds. Velmans M. Schneider S. (Oxford: Blackwell), 657–670.

[ref9] Bayne T. Seth A. K. Massimimi M. (2020). Are there islands of awareness? Trends Neurosci. 43, 6–16. doi: 10.1016/j.tins.2019.11.003 31836316

[ref10] Berridge K. C. Kringelbach M. L. (2015). Pleasure systems in the brain. Neuron 86, 646–664. doi: 10.1016/j.neuron.2015.02.018, PMID: 25950633 PMC4425246

[ref11] Blumenfeld H. (2023). Brain mechanisms of conscious awareness: detect, pulse, switch, and wave. Neuroscientist 29, 9–18. doi: 10.1177/10738584211049378, PMID: 34632846 PMC8995398

[ref12] Bressler S. (2008). Neurocognitive networks. Scholarpedia 3:1567. doi: 10.4249/scholarpedia.1567

[ref13] Budson A. E. Richman K. A. Kensinger E. A. (2022). Consciousness as a memory system. Cogn. Behav. Neurol. 35, 263–297. doi: 10.1097/WNN.0000000000000319, PMID: 36178498 PMC9708083

[ref14] Cabanac M. Cabanac J. Parent A. (2009). The emergence of consciousness in phylogeny. Behav. Brain Res. 198, 267–272. doi: 10.1016/j.bbr.2008.11.028, PMID: 19095011

[ref15] Chalmers D. J. (1995). Facing up to the problem of consciousness. J. Cons. Stud 2, 200–219. doi: 10.1093/acprof:oso/9780195311105.003.0001

[ref16] Chalmers D. J. (2003) in “Consciousness and its place in nature” in Blackwell guide to the philosophy of mind. eds. Stich S. P. Warfield T. A. (Oxford: Oxford University Press), 102–142.

[ref17] Cosmides L. Tooby J. (1994). “Origins of domain specificity: the evolution of functional organization” in Mapping the mind: Domain specificity in cognition and culture. eds. Hirschfeld L. A. Gelman S. A. (Cambridge: Cambridge University Press), 85–116.

[ref18] De Stefani E. De Marco D. (2019). Language, gesture, and emotional communication: an embodied view of social interaction. Front. Psychol. 10:2063. doi: 10.3389/fpsyg.2019.02063, PMID: 31607974 PMC6769117

[ref19] Dennett D. C. (1991). Consciousness explained. New York: Little, Brown and Co.

[ref20] Earl B. (2014). The biological functions of consciousness. Front. Psychol. 5:697. doi: 10.3389/fpsyg.2014.00697, PMID: 25140159 PMC4122207

[ref21] Earl B. (2019). The structure of mind and the role of consciousness. J. Psychol. Behav. Sci. 7, 84–101. doi: 10.15640/jpbs.v7n2a9

[ref22] Edwards J. Somov P. G. (2023). The case for conscious experience being in individual neurons. Qeios preprint. doi: 10.32388/DEUK7V.4

[ref23] Feinberg T. E. (2024). From sensing to sentience: How feeling emerges from the brain. Cambridge, MA: MIT Press.

[ref24] Gennaro R. J. (Ed.)(2018). “Representational theories of consciousness” in The Routledge handbook of consciousness (London: Taylor & Francis), 107–121.

[ref25] Gentilucci M. Corballis M. C. (2006). From manual gesture to speech: a gradual transition. Neurosci. Biobehav. Rev. 30, 949–960. doi: 10.1016/j.neubiorev.2006.02.004, PMID: 16620983

[ref26] Goff P. (2006). Experiences don’t sum. J. Cons. Stud. 13, 53–61.

[ref27] Goff P. (2009). Why panpsychism doesn’t help explain consciousness. Dialectica 69, 289–311. doi: 10.1111/j.1746-8361.2009.01146.x

[ref28] Gomez-Marin A. Seth A. K. (2025). A science of consciousness beyond pseudo-science and pseudo-consciousness. Nat. Neurosci. 28, 703–706. doi: 10.1038/s41593-025-01913-6, PMID: 40065186

[ref29] Grandin T. (1996). Thinking in pictures: My life with autism. New York: Doubleday.

[ref30] Hales C. G. Ericson M. (2022). Electromagnetism’s bridge across the explanatory gap: how a neuroscience/physics collaboration delivers explanation into all theories of consciousness. Front. Hum. Neurosci. 16:836046. doi: 10.3389/fnhum.2022.836046, PMID: 35782039 PMC9245352

[ref31] Han K. S. Guo C. Chen C. H. Witter L. Osorno T. Regehr W. G. (2018). Ephaptic coupling promotes synchronous firing of cerebellar Purkinje cells. Neuron 100, 564–578.e3. doi: 10.1016/j.neuron.2018.09.018, PMID: 30293822 PMC7513896

[ref32] Hunt T. Jones M. (2023). Fields or firings? Comparing the spike code and the electromagnetic field hypothesis. Front. Psychol. 14:1029715. doi: 10.3389/fpsyg.2023.1029715, PMID: 37546464 PMC10400444

[ref33] Hunt T. Schooler J. W. (2019). The easy part of the hard problem: a resonance theory of consciousness. Front. Hum. Neurosci. 13:378. doi: 10.3389/fnhum.2019.00378, PMID: 31736728 PMC6834646

[ref34] Irwin L. N. (2023). What current theories of consciousness are missing. Neurol. Neurosci. 4, 1–4. doi: 10.33425/26927918.1059

[ref35] Ito T. Yang G. R. Laurent P. Schultz D. H. Cole M. W. (2022). Constructing neural network models from brain data reveals representational transformations linked to adaptive behavior. Nat. Commun. 13:673. doi: 10.1038/s41467-022-28323-7 35115530 PMC8814166

[ref36] Jackendoff R. S. (2002). Foundations of language: Brain, meaning, grammar, evolution. Oxford: Oxford University Press. 10.1017/s0140525x03000153 15377127

[ref37] Kitchener P. D. Hales C. G. (2022). What neuroscientists think, and don’t think, about consciousness. Front. Hum. Neurosci. 16:767612. doi: 10.3389/fnhum.2022.767612, PMID: 35280212 PMC8907974

[ref38] Lacalli T. C. (2020). Evolving consciousness: insights from Turing, and the shaping of experience. Front. Behav. Neurosci. 14:598561. doi: 10.3389/fnbeh.2020.598561, PMID: 33328924 PMC7719830

[ref39] Lacalli T. C. (2021). Consciousness as a product of evolution: contents, selector circuits, and trajectories in experience space. Front. Syst. Neurosci. 15:697129. doi: 10.3389/fnsys.2021.697129, PMID: 34744646 PMC8564397

[ref40] Lacalli T. C. (2022). On the origins and evolution of qualia: an experience-space perspective. Front. Syst. Neurosci. 16:945722. doi: 10.3389/fnsys.2022.945722, PMID: 36032325 PMC9399462

[ref41] Lacalli T. C. (2024). The function(s) of consciousness: an evolutionary perspective. Front. Psychol. 15:1493423. doi: 10.3389/fpsyg.2024.1493423, PMID: 39660268 PMC11628302

[ref42] Levine J. (1983). Materialism and qualia: the explanatory gap. Pac. Phil. Quart. 64, 354–361.

[ref43] Levine J. (2009). “The explanatory gap” in The Oxford handbook of philosophy of mind. eds. Beckerman A. McLaughlin B. P. Walter S. (Oxford: Oxford University Press), 281–291.

[ref44] Lewin R. (1984). Why is development so illogical? Science 224, 1327–1329. 6374894 10.1126/science.6374894

[ref45] Majeed R. (2016). The hard problem and its explanatory targets. Ratio 29, 298–311. doi: 10.1111/rati.12103

[ref46] Manzotti R. (2012). The computational stance is unfit for consciousness. Int. J. Mach. Constr. 4, 401–420. doi: 10.1142/S1793843012400239

[ref47] Merker B. (2007). Consciousness without a cerebral cortex: a challenge for neuroscience and medicine. Behav. Brain Sci. 30, 63–81. doi: 10.1017/S0140525X07000891, PMID: 17475053

[ref48] Merker B. (2013). The efference cascade, consciousness, and its self: naturalizing the first person pivot of action control. Front. Psychol. 4:50. doi: 10.3389/fpsgy.2013.00501 23950750 PMC3738861

[ref49] Montero B. G. (2016). “What combination problem?” in Panpsychism: Contemporary perspectives. eds. Brüntrup G. Jaskolla L. (Oxford, UK: Oxford University Press), 215–228.

[ref50] Morsella E. Godwin C. A. Jantz T. K. Krieger S. C. (2016). Homing in on consciousness in the nervous system: an action-based synthesis. Behav. Brain Sci. 39:e168. doi: 10.1017/S0140525X15000643 26096599

[ref51] Neafsey E. J. (2021). Conscious intention and human action: review of the rise and fall of the readiness potential and Libet’s clock. Cons. Cogn. 94:103171. doi: 10.1016/j.concog.2021.103171 34325185

[ref52] Overgaard M. (2018). Phenomenal consciousness and cognitive access. Philos. Trans. R. Soc. Lond. Ser. B Biol. Sci. 373:20170353. doi: 10.1098/rstb.2017.0353, PMID: 30061466 PMC6074085

[ref53] Petersen S. Sporns O. (2015). Brain networks and cognitive architecture. Neuron 88, 207–219. doi: 10.1016/j.neuron.2015.09.027 26447582 PMC4598639

[ref54] Pinker S. Jackendoff R. S. (2005). The faculty of language: what’s special about it? Cognition 95, 201–236. doi: 10.1016/j.cognition.2004.08.004, PMID: 15694646

[ref55] Pockett S. (2012). The electromagnetic field theory of consciousness. J. Cons. Stud. 19, 191–223.

[ref56] Polák M. Marvan T. (2019). How to mitigate the hard problem by adapting the dual theory of phenomenal consciousness. Front. Psychol. 10:2837. doi: 10.3389/fpsyg.2019.02837 31920868 PMC6927938

[ref57] Robbins P. (2017). “Modularity of mind” in The Stanford encyclopedia of philosophy, ed Zalta E. N. (Stanford, CA: Metaphysics Research Lab, Stanford University). Available online at: https://plato.stanford.edu/archives/win2017/entries/modularity-mind/

[ref58] Robinson D. G. Mallatt J. Peer W. A. Sourjik V. Taiz L. (2024). Cell consciousness: a dissenting opinion. EMBO Rep. 25, 2162–2167. doi: 10.1038/s44319-024-00127-4, PMID: 38548972 PMC11094104

[ref59] Ruffini G. Salvador R. Tadayon E. Sanchez-Todo R. Pascual-Leone A. Santarnecchi E. (2020). Realistic modeling of mesoscopic ephaptic coupling in the human brain. PLoS Comput. Biol. 16:e1007923. doi: 10.1371/journal.pcbi.1007923, PMID: 32479496 PMC7289436

[ref60] Sauseng P. Klimesch W. (2008). What does phase information of oscillating brain activity tell us about cognitive processes? Neurosci. Biobehav. Rev. 32, 1001–1013. doi: 10.1016/j.neubiorev.2008.03.014, PMID: 18499256

[ref61] Seth A. K. Bayne T. (2022). Theories of consciousness. Nat. Rev. Neurosci. 23, 439–452. doi: 10.1038/s41583-022-00587-4, PMID: 35505255

[ref62] Stinson C. (2018). “Explanation and connectionist models” in The Routledge handbook of the computational mind. eds. Sprevak M. Colombo M. (London: Taylor & Francis), 120–133.

[ref63] Sun R. Franklin S. (2007). “Computational models of consciousness: a taxonomy and some examples” in The Cambridge handbook of consciousness. eds. Zelazo P. D. Moscovitch M. Thompson E. (Cambridge, UK: Cambridge University Press), 151–174.

[ref64] Tacikowski P. Kalender G. Citiberti D. Fried I. (2024). Human hippocampal and entorhinal neurons encode the temporal structure of experience. Nature 635, 160–167. doi: 10.1038/s41586-024-07973-1 39322671 PMC11540853

[ref65] Triggiani A. I. Kreiman G. Lewis C. Maoz U. Mele A. Mudrik L.. (2023). What is the intention to move and when does it occur? Neurosci. Biobehav. Rev. 151:105199. doi: 10.1016/j.neubiorev.2023.105199 37119992 PMC10330627

[ref66] Varela F. Lachaux J. P. Rodriguez E. Martinerie J. (2001). The brainweb: phase synchronization and large-scale integration. Nat. Rev. Neurosci. 2, 229–239. doi: 10.1038/35067550, PMID: 11283746

[ref67] Weiss S. A. Faber D. S. (2010). Field effects in the CNS play functional roles. Front. Neural Circuits 4:15. doi: 10.3389/fncir.2010.00015 20508749 PMC2876880

[ref68] Wood C. C. (2019). The computational stance in biology. Philos. Trans. R. Soc. Lond. Ser. B Biol. Sci. 374:20180380. doi: 10.1098/rstb.2018.0380, PMID: 31006370 PMC6553588

[ref69] Zeki S. (2003). The disunity of consciousness. Trends Cogn. Sci. 7, 214–218. doi: 10.1016/S1364-6613(03)00081-0, PMID: 12757823

